# Effect of High-Pressure Torsion on Structure and Properties of Ti-15Mo/TiB Metal-Matrix Composite

**DOI:** 10.3390/ma11122426

**Published:** 2018-11-30

**Authors:** Sergey Zherebtsov, Maxim Ozerov, Margarita Klimova, Nikita Stepanov, Tatyana Vershinina, Yulia Ivanisenko, Gennady Salishchev

**Affiliations:** 1 Laboratory of Bulk Nanostructured Materials, Belgorod State University, Belgorod 308015, Russia; ozerov@bsu.edu.ru (M.O.); klimova_mv@bsu.edu.ru (M.K.); stepanov@bsu.edu.ru (N.S.); salishchev@bsu.edu.ru (G.S.); 2 Centre of Nanostructure Materials and Nanotechnology, Belgorod State University, Belgorod 308015, Russia; vershinina@bsu.edu.ru; 3 Karlsruhe Institute of Technology, Institute of Nanotechnology, Karlsruhe 76021, Germany; julia.ivanisenko@kit.edu

**Keywords:** metal-matrix composite, titanium, high-pressure torsion, nanostructure, microstructure evolution, microhardness

## Abstract

The microstructure and microhardness evolution of a Ti-15(wt.%)Mo/TiB metal-matrix composite (MMC) during high-pressure torsion (HPT) at 400 °C was studied. The composite was fabricated by spark plasma sintering of a Ti, Mo and TiB_2_ powders mixture at 1200 °C. In the initial condition, the structure of the composite consisted mainly of body-centered cubic (bcc) Ti solid solution and TiB whiskers. An increase in dislocation density, a considerable decrease in a grain size in the bcc Ti matrix, and breaking/rearrangement of the TiB whiskers were observed during HPT. The (sub)grain size in the bcc Ti matrix attained after 1 revolution was ~75 nm and then gradually decreased to ~55 nm after 5 revolutions. The TiB particle sizes after 5 revolutions was found to be 130–210 nm. The microhardness increased with strain from 575 HV in the initial state to 730 HV after 5 revolutions. Various hardening mechanisms’ contributions in the Ti-15Mo/TiB were evaluated.

## 1. Introduction

Titanium and some titanium alloys are used in medicine to a great extent due to good corrosion resistance, high specific strength and excellent biocompatibility. Surgical implants and armatures made of titanium are light, do not corrode in biological fluids and do not cause allergic reactions [1]. However absolute values of hardness and yield strength of titanium alloys is rather low [2] and limits their use for same specific applications, for example fabrication of scalpels, operating knives or scissors. Production of metal-matrix composites (MMCs) by embedding rigid ceramic particles into the matrix is a promising way to improve the strength and hardness of titanium [3]. Among various reinforcements TiB has a close to titanium density and creates low residual stresses due to a good crystallographic match with the matrix [4,5]. The Ti/TiB specimens can be produced during the spark plasma sintering (SPS) process via the TiB_2_ + 2Ti → Ti + 2TiB in situ reaction [6]. However, the obtained values of strength and hardness of hexagonal close-packed (hcp) Ti/TiB MMCs were found to be insufficiently high to use this material for production of surgical cutting instruments [7,8].

One of the possible options to overcome this problem can be associated with the changing of matrix structure from the hcp lattice to the body-centered cubic (bcc) one via addition of a beta stabilizer(s) like, for example, Mo. In particular, a bcc Ti-15Mo alloy is widely used in medicine due to high specific strength, excellent biocompatibility and low Yong’s modulus. In addition, the strength and hardness of β titanium alloys with a (meta)stable bcc structure can be considerably enhanced by thermomechanical treatment [9].

Some mechanical properties of alloys can be modified substantially using severe plastic deformation (SPD) [10] due to refinement of their microstructure down to the nanoscale interval. Although different SPD methods was a subject of comprehensive investigations during recent decades [10,11,12], the influence of severe deformation on the structure and properties of MMCs has been poorly studied yet [13,14]. Meanwhile, the SPD of such types of materials is expected to refine the matrix microstructure, decrease the size of particles and improve the particle distribution; the operation of several strengthening mechanisms can result in a record level of strength and hardness in severely deformed MMCs [13,14,15]. Since the ductility of MMCs is usually not high enough, the most suitable SPD process seems to be high-pressure torsion (HPT) at elevated temperature, which was successfully used earlier for nanostructuring of various MMCs, including hcp Ti/TiB [14]. It should be mentioned, however, that examinations of MMC microstructure evolution during SPD were mainly carried out using composites with oxide or carbon-based equiaxial particles [12,13]. Composites reinforced by fibers or elongated particles, like those formed in the Ti/TiB system [8,14], have been studied to a much lesser extent. Meanwhile (to the best of the authors’ knowledge) the structure and mechanical properties of bcc-Ti based MMCs subjected to HPT has not been studied so far.

In the present work, a Ti-15Mo/TiB composite was fabricated through SPS using a Ti-13.5(wt.%) Mo-10(wt.%) TiB_2_ powder mixture at 1200 °C. The composite was then deformed using high-pressure torsion at 400 °C to different strain levels. The microstructure evolution of the composite was comprehensively studied using X-ray diffraction (XRD), scanning electron microscopy (SEM) and transmission electron microscopy (TEM); microhardness was measured to evaluate the influence of HPT on its mechanical properties.

## 2. Experimental

Powders of commercial purity Ti (99.1% purity), Mo (99.95% purity) and TiB_2_ (99.9% purity) were (Guangzhou Hongwu Material Technology Co., Ltd., Guangzhou, China) used for the sintering. The average sizes of the Ti, Mo and TiB_2_ particles in the powders were 25, 20 and 4 μm, respectively. A powders mixture contained 86.5 wt.% Ti, 13.5 wt.% of Mo and 10 wt.% of TiB_2_ was produced (to obtain a Ti-15wt.% Mo alloy with 17 vol.% of TiB) using a Retsch RS200 vibrating cup (RETSCH Technology, Haan, Germany) mill in ethanol for 1h; a milling rotation speed was 700rpm. Specimens measured Ø19 mm × ~20 mm height of Ti-15Mo/TiB MMC were produced by SPS at 1200 °C and 40 MPa under vacuum for 5 min on a Thermal Technology SPS10-3 set-up (Thermal Technology LLC, Santa Rosa, CA, USA).

Disks 10 mm in diameter and 0.7 mm in thickness were strained by HPT in a Bridgman-type anvil unit equipped with a custom-built computer-controlled device (W. Klement GmbH, Lang, Austria) at 400 °C with a speed of 1 rpm and load of 6 GPa. The quantity of turns was 1, 3 or 5. Shear strain γ for the corresponding number of revolutions can be evaluated as [12]:

(1) $$\gamma =\frac{2\pi \mathrm{Nr}}{h}$$

where N is the number of revolutions, h is the thickness and r is the radius of the specimen. The elevated deformation temperature was selected to ensure enough ductility during HPT.

The XRD study was done using an ARL-Xtra diffractometer (Thermo Fisher Scientific, Waltham, MA, USA) with CuKα radiation. Dislocation density ρ, was evaluated using the following equation [16]:

(2) $$\rho =\frac{3\sqrt{2\pi }\langle \varepsilon_{50}^{2}\rangle }{\mathrm{Db}}$$

where b is the the Burgers vector; the microstrains $\langle \varepsilon_{50}^{2}\rangle$ and size of crystallites D were evaluated per the Williamson–Hall method [17]:

(3) $$\frac{\beta_{s}\cos\theta }{\lambda }=\frac{2\langle \varepsilon_{50}^{2}\rangle \sin\Theta }{\lambda }+\frac{K}{D}$$

where β_s_ denotes the corrected full width at the half maximum (FWHM) of proper Kα_1_ reflections, Θ denotes the Bragg angle of the corresponding peaks, λ is the Kα_1_ wavelength, K is the Scherrer constant. The determined (110)β peaks positions and the FWHM values were used for the computations. The instrumental broadening was evaluated using the values of FWHM for annealed silicon powders. Quantitative analysis of phase composition was performed per the Rietveld method [18] using PowderCell software.

A Quanta 600 FEG scanning-electron microscope (SEM) (FEI, Lausanne, Switzerland) equipped with an energy-dispersive spectrometry (EDS) (Thermo Fisher Scientific, Waltham, MA, USA) detector for chemical analysis and a JEOL JEM-2100 transmission electron microscope (TEM) (JEOL Ltd., Tokyo, Japan) were used for structure investigation. SEM analysis and microhardness measurements were studied in the axial cross-section at a distance of 0; 2.5 or 5 mm from the center. TEM observations were done in the shear plane at ~1.5 mm from the disc edge. 

SEM specimens were mechanically polished and etched with Kroll’s reagent (95% H_2_O, 3% HNO_3_, 2% HF). Samples for TEM analysis were prepared by twin-jet electro-polishing in a mixture of 60 mL perchloric acid, 600 mL methanol and 360 mL butanol at −35 °C and 29 V.

Vickers microhardness was measured with 1 kG load for 10 s. The values of hardness were averaged over at least 10 individual measurements per each data point.

## 3. Results

**Figure 1:**
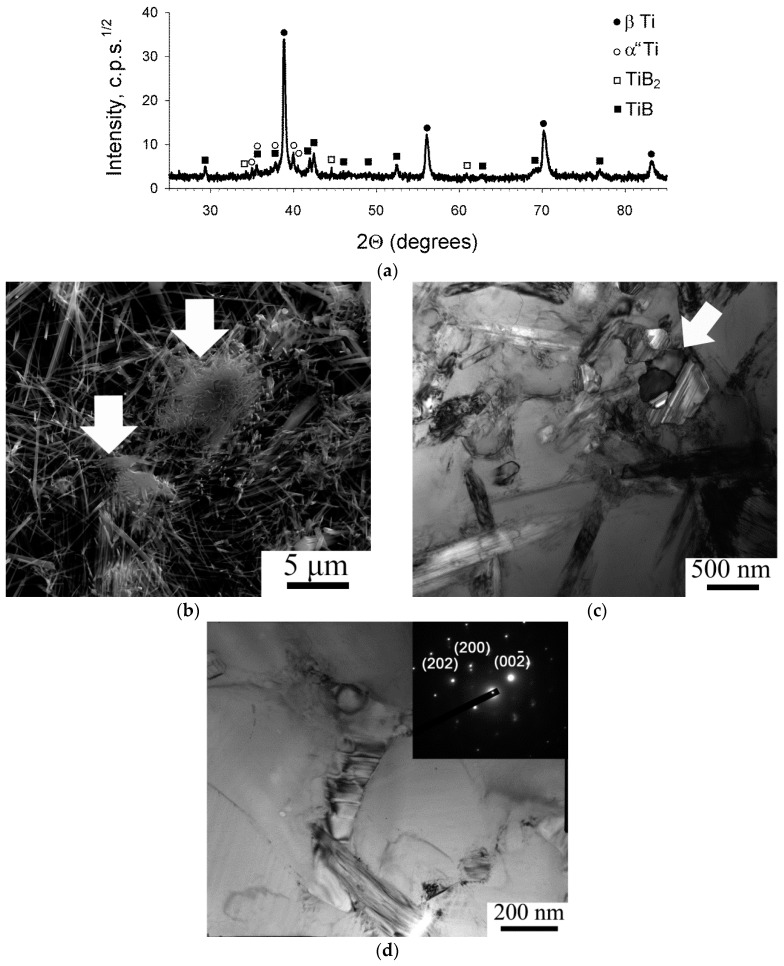
Phase composition (**a**) and microstructure of Ti-15Mo/TiB composite produced at 1200 °C: (**b**) scanning electron micrograph (SEM), etched surface; (**c**,**d**) bright-field transmission electron micrograph (TEM).

The XRD patterns showed (Figure 1a) that the Ti-15Mo/TiB composite sintered at 1200 °C in the initial condition consisted of β-Ti solid solution (bcc lattice), α” martensite (orthorhombic lattice), TiB (orthorhombic lattice), and retained TiB2 (hexagonal lattice). The quantitative analysis revealed the presence of 84% β-Ti, 2% TiB2, 10% TiB and 4% α”.

Analysis of the Ti-15%Mo/TiB composite microstructure in the as-sintered condition revealed TiB fibers (whiskers) non-uniformly distributed in the bcc Ti matrix and some particles of residual TiB_2_ (marked in Figure 1b by arrows). The average diameter of TiB fibers was found to be 130 nm; however, very thick TiB whiskers of ~1–2 µm can also be observed in the microstructure. The TiB whiskers in the bcc Ti matrix can also be seen in TEM images (Figure 1c). Dislocation density was generally rather high; however, in some places individual TiB whiskers can be clearly seen. Grain boundaries cannot be distinguished in the bcc Ti matrix; however, spacing between the TiB whiskers (which to a first approximation can be considered as a free dislocation path) was ~1–1.5 µm. 

The matrix bcc phase had some variations in the content of Mo in the interval ~8–15 wt.% (at the nominal concentration in the composition 13.5 wt.%) without any visible phase separation. Small elongated particles of the α” phase were observed in some places (Figure 1d) in agreement with the XRD results (Figure 1a). The ω phase was detected by neither Energy Dispersive X-ray Analysis (EDX) nor TEM that seems to be rather typical of β-Ti alloys produced by SPS [19]. 

The TiB whiskers had a hexagonal-like shape (shown by an arrow in Figure 1c) in which planes (100), (101) and ($10\bar{1}$) formed along the whiskers sides [20]. A lot of stacking faults formed in the plane parallel to (100). The Ti/TiB interfaces were found to be quite clear due to low internal stresses nearby the interphase boundaries. The latter most likely resulted from the orientation relationship (OR) between the Ti matrix and the TiB particles (known in the form: (110_β_//(001)_TiB_ and [$\bar{1}11$]_β_//[010]_TiB_ [6]).

**Figure 2:**
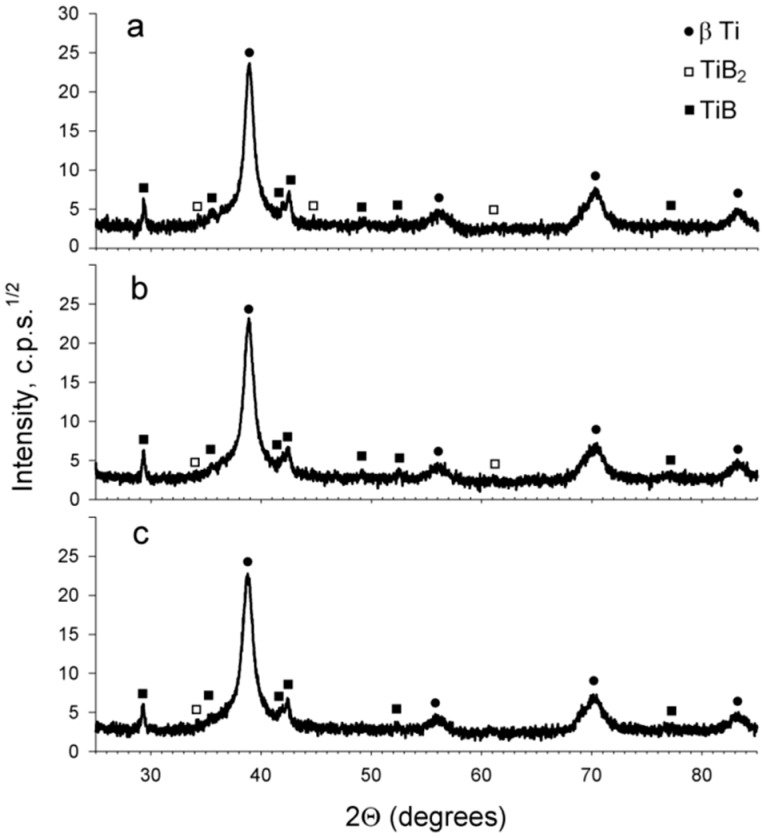
X-ray diffraction (XRD) patterns of the Ti-15Mo/TiB composite after: (**a**) 1; (**b**) 3; (**c**) 5 revolutions of high-pressure torsion (HPT).

XRD patterns obtained after 1, 3 or 5 revolutions of HPT (Figure 2) showed a vanishing of peaks that belonged to the martensitic phase and some decrease in the TiB_2_ phase peak intensity. The content of bcc Ti increased by ~4% during deformation most likely due to α” dissolution. All diffraction maximums became broader in the deformed conditions which can be associated with a lower crystallite size and higher lattice microstrains.

Indeed, quantitative analysis of the XRD data (Table 1) showed a considerable decrease in a crystallite size and an increase in the level of lattice microstrains in deformed specimens as compared to those in the initial condition. At the same time, an increase in the quantity of turns from 1 to 5 decreased both microstrains and crystallite size. Note that dislocation density behaved similarly to the microstrain, i.e., it significantly increased at the beginning of the HPT process and then gradually decreased. This type of behavior can be associated with reorganization of dislocations and gradual transformation of deformation cells into (sub)grains with moderate-to-high misorientations [21].

**Figure 3:**
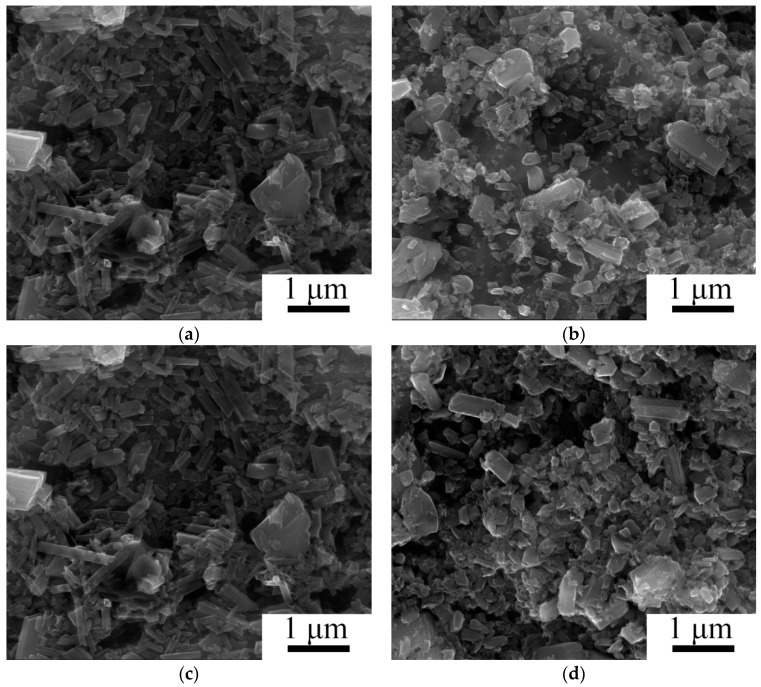
SEM images of etched surfaces of Ti-15Mo/TiB specimens after HPT: (**a**,**c**) in the center; (**b**,**d**) at the edge; (**a**,**b**) 1 revolution; (**c**,**d**) 5 revolutions.

Evolution of the composite microstructure during HPT expectably depended on the spacing from the specimen center (Figure 3). After 1 revolution of HPT, the microstructure changes in the central part of the specimen were mainly associated with fracturing of the TiB whiskers (Figure 3a). Near the specimen edge (γ = 45) the debris of TiB whiskers were much finer; in addition, some tiny grains (less than 100 nm) can be seen at the etched surface of the matrix indicating the onset of nano-sized grains formation in bcc Ti (Figure 3b). Microstructure refinement gradually developed with strain both in the center and at the edge of the specimen resulting in the formation of quite a uniform microstructure after 5 revolutions of HPT (Figure 3c,d). However, TiB debris at the edge of the specimen after severe deformation (γ = 224) was finer and had a more equiaxial shape (Figure 3d).

**Figure 4:**
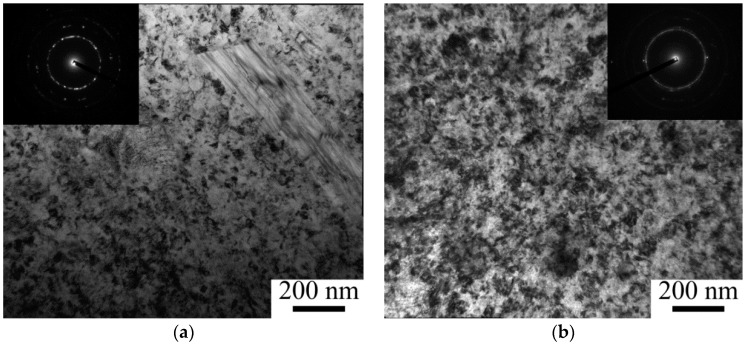
Bright-field TEM images of the Ti-15Mo/TiB microstructure as a result of: (**a**) 1; (**b**) 5 revolutions of HPT.

**Figure 5:**
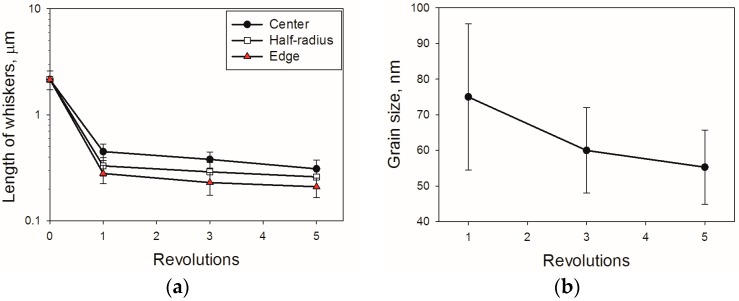
Influence of HPT on: (**a**) apparent length of TiB fibers in Ti-15Mo/TiB; (**b**) (sub)grain size in the bcc titanium matrix.

TEM examination also confirmed substantial refinement of the bcc MMC matrix due to HPT (Figure 4). One revolution (γ = 31) formed a microstructure composed of tiny (~75 nm) (sub)grains and dislocation pile-ups (Figure 4a, Figure 5b). The interphase boundaries between bcc Ti and TiB become unclear because of high internal stresses which can be associated with the high dislocation density in the matrix; however, no voids or cracks were observed at the interfaces. There were no noticeable qualitative changes in the microstructure at larger deformation, however a decrease in the (sub)grain size to ~60 and ~50 nm was observed with increasing strain to γ = 94 (3 revolutions) and to γ = 157 (5 revolutions), respectively (Figure 4b, Figure 5b). In addition, the formation of a ring-like diffraction pattern after 5 revolutions suggests evolution of a subgrain structure into a grain one with strain (inserts in Figure 4a,b). These results correlate with decreasing crystalline size; the ratio between the (sub)grain size and the crystalline size was ~1.5–2.5/1 (Table 1). 

A uniform distribution of Mo in the bcc matrix was detected as a result of 5 revolutions of HPT; a similar effect of severe deformation (cold-consolidation of metallic powders into bulk samples with homogeneous elements’ distribution using HPT) was reported earlier e.g., [12]. Although plastic deformation at 400 °C can potentially result in the β→ω transformation [22], the ω phase after 1–5 turns of HPT was not found. This result agrees with some earlier investigations showing the development of the opposite ω→β transformation during HPT of bcc Ti alloys [23]. However it is worth noting that the ω phase formation in the beginning of HPT of the Ti-15Mo alloy at room temperature was observed in [24].

The apparent length of the TiB fibers nearby the specimen edge decreased in ~7.5 times as a result of 1 HPT turn and then declined gradually (by ~33%) with an increase in turns from 1 to 5 (Figure 5a). Similar behavior demonstrated TiB whiskers in the center or at the half-radius of the specimen; however, the corresponding absolute values were 1.5–2 times higher in comparison with the TiB length at the edge of the specimen. The diameter of the whisker was not affected noticeably by HPT. The aspect ratio (length/diameter) of the TiB fibers reached values of ~1.5 (i.e., they can be considered nearly equiaxed) at N = 5. For example, the aspect ratio in the initial condition was ~16.

**Figure 6:**
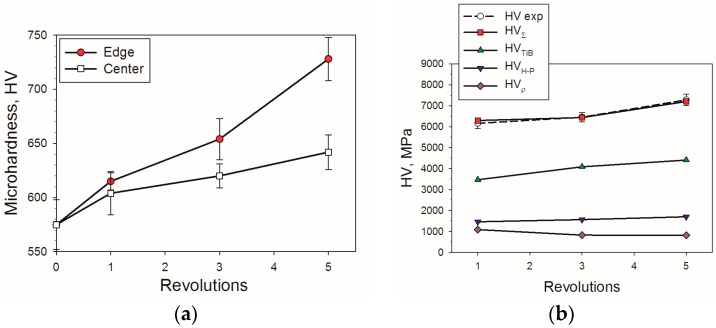
Microhardness of the Ti-15Mo/TiB as a function of HPT turns: (**a**) experimental data; (**b**) various hardening mechanisms’ contribution to overall hardness (data taken for the specimen edge).

Figure 6 shows the microhardness evolution of the Ti-15%Mo/TiB MMC as a result of severe deformation. The microhardness of the specimen nearby the edge increased during HPT faster than that in the center. It should be noted that the difference between the hardness of the central part and edge increased with strain. The maximum microhardness of ~730 HV and 640 HV at the edge and in the center, respectively, was reached after 5 revolutions. The values obtained were noticeably greater than those in the as-sintered state (575 HV); the increase was ~11% for the central part and ~27% for the specimen edge. Basically, the hardness at the edge and in the central part of the specimens gradually becomes equal to each other during HPT [12]. In our case the level of strain was most likely insufficient to homogenize the microstructure and microhardness along the diameter.

Comparing the obtained data for the microhardness of hcp Ti/TiB with the same amount of the reinforcement after HTP at the same temperature [14], one can see that the change of the matrix structure from the hcp lattice to the bcc one yields ~13% hardening in the initial condition (575 HV and 450 HV for bcc and hcp Ti, respectively) and ~43% hardening after 5 revolutions of HPT (730 HV and 510 HV, respectively).

## 4. Discussion

The results obtained show the possibility to produce a Ti-15Mo/TiB metal-matrix composite specimen using SPS of a multicomponent mixture of elemental powders and involving the TiB_2_ + 2Ti → Ti + 2TiB in situ chemical reaction. HPT results in a uniform distribution of alloying elements in the microstructure due to a deformation-driven mechanical alloying and structure refinement at the nanometer scale.

Nanocrystalline microstructure formation in different metals and alloys during HPT has been reported comprehensively [12]; some studies focused on metal-matrix composites [13]. Generally, structure evolution caused by HPT can be described in terms of classical models of structure transformations taking place during usual plastic metal working [25,26]. However, due to high applied hydrostatic pressure which prevents breaking of the specimens even during very intensive staining the processes of structure formation occurs much faster and can result in the formation of a very small grain size [10,11,12]. Indeed, a uniform microstructure with the size of grains of 55 nm was formed in Ti-15Mo/TiB after 5 revolutions of HPT. Although an increase in the processing temperature usually results in greater deformation-induced grains, in our case the grain size in the HPT-ed Ti-15Mo/TiB at 400 °C was approximately two times smaller than that observed in Ti-15%Mo alloys after HPT at room temperature (~90 nm after 10 revolutions [24]). The formation of the finer microstructure can be ascribed essentially to constrained deformation because of a very high dislocation density and many TiB whiskers in the as-sintered state. Most likely the size of grains d which form during deformation depended on the corresponding flow stress σ_s_ as per the σ_s_ = Kd^−N^ relation, where N and K are constants [27]. The value of σ_s_ increases in case of constrained deformation in MMCs, thereby producing smaller grain size.

It worth noting that the size of (sub)grains which form in the MMC based on hcp commercially pure Ti during the same SPD processing (HPT at 400 °C) was considerably smaller (~30 nm after 5 revolutions) [14]. However, in the case of “pure alloys” (i.e., without reinforcements) the grain size obtained in hcp Ti during HPT (100–130 nm after 8–10 turns) [28,29,30,31] was only slightly greater than that observed in the Ti-15Mo alloy [23,24], suggesting that the final microstructure does not depend considerably on the lattice type. 

Another effect of SPD on the Ti-15Mo/TiB MMC microstructure was associated with almost an order of magnitude decrease in the TiB fibers’ length and a uniform TiB debris redistribution in the bcc titanium matrix. As discussed in [32] a decrease in the aspect ratio of elongated reinforcements to the values below ~10 should result in a considerable increase in the Orowan strengthening contribution, thereby giving rise to a noticeable increase in strength of a MMC.

Indeed, the observed decrease in both the grain size and length of the TiB fibers led to the MMC hardness increase (Figure 6a). The contributions of the main strengthening mechanisms to the overall composite hardness ${\mathrm{HV}}_{\Sigma }$ can be expressed as:

(4) $${\mathrm{HV}}_{\Sigma }={\mathrm{HV}}_{0}+{\mathrm{HV}}_{\rho }+{\mathrm{HV}}_{H-P}+{\mathrm{HV}}_{\mathrm{TiB}}$$

where ${\mathrm{HV}}_{0}$ includes Peierls stress and solid solution strengthening, ${\mathrm{HV}}_{\rho }$ is a strength increment caused by increased dislocation density (substructure hardening), ${\mathrm{HV}}_{H-P}$ denotes grain-size (Hall–Petch) hardening and ${\mathrm{HV}}_{\mathrm{TiB}}$ is precipetation hardening by the debris of TiB. The contribution of substructure hardening ${\mathrm{HV}}_{\rho }$ can be described as [33,34]:

(5) $${\mathrm{HV}}_{\rho }=M\alpha \mathrm{Gb}\sqrt{\rho }$$

where M is the Taylor factor (a typical value of M for the bcc structure is 3 [35]), α is a constant (α = 0.2 was accepted for the current calculation), G is the shear modulus, ρ is dislocation density and b is the Burgers vector. The contribution of the Hall–Petch strengthening can be described by the formula [36,37]:

(6) HVH−P=Kyd−12

in which K_y_ denotes the Hall–Petch coefficient, d—the size of (sub)grains. The precipitation hardening ${\mathrm{HV}}_{\mathrm{TiB}}$ can be calculated as [38]:

(7) HVTiB=(0.538Gbf12X)ln(X2b)

where X and f are the diameter and the volume fraction of TiB particles, respectively.

For the bcc Ti input parameters were adopted as follows: K_y_ = 0.4 MPa m^0.5^ [39], HV_0_ = 320 MPa [24] and b = 2.86 × 10^−10^ m [40]. The shear stress G = 31.8 GPа was obtained using data from [4,24]. Data from Figure 5 and Table 1 were used to determine the size of (sub)grains d as well as particles diameter X and dislocation density ρ. The volume fraction f = 0.1 of TiB determined using XRD analysis. The net hardness as a sum of various hardening mechanisms is shown in Figure 6b. The main contribution to the overall hardness can be expected from the TiB particles hardening (twice as high as the rest of the sources combined). This contribution increased during HPT due to the TiB particles’ refinement [32,38]. The uniform distribution of debris in the TiB can result in an increase in ductility [13,41,42]; however, further investigations are needed to establish this effect more reliably. Substructure hardening makes the smallest contribution to hardness which became even smaller during the severe plastic deformation processing due to the decrease in dislocation density (Table 1). The Hall–Petch strengthening gives a somewhat higher contribution in comparison to that of ${\mathrm{HV}}_{\rho }$. Due to the microstructure refinement, the Hall–Petch hardening slightly increased during HPT.

It can be concluded, therefore, that the experimental results obtained can be described quite accurately as the sum of the main strengthening mechanisms. The most important contribution of precipitation hardening in comparison with the substructure and grain-size (Hall–Petch) hardenings should be noted. This finding confirms that the MMCs properties are most likely determined by morphology, size and distribution of the reinforced particles rather than with properties of the matrix. It should be noted also that the obtained maximum microhardness in the MMC was ~730HV (Figure 6a); this value is equivalent to ~58.5HRC which, in turn, corresponds to the hardness of a quenched steel (55-60HRC) usually used for production of cutting instruments [43]. Therefore, the Ti-15Mo/TiB MMC subjected to large straining can be considering as a promising material for the production of surgical cutting instruments.

## 5. Conclusions

The evolution of microstructure and microhardness of Ti-15Mo/TiB metal-matrix composite produced by SPS at 1200 °C were investigated during HPT at 400 °C. Several conclusions can be drawn from this work:

(1) The Ti-15Mo/TiB composite fabricated by SPS was composed of the matrix (84%) bcc β-Ti, 4% of the orthorhombic α” martensite, 10% of the TiB whiskers, and 2% of unreacted TiB_2_. The TiB whiskers had an average diameter of 130 nm and aspect ratio of ~16. Also, some variation in the local concentration of Mo in the bcc Ti matrix was found.

(2) The microstructure evolution of the Ti-15Mo/TiB composite during HPT was associated with breaking/rearrangement of TiB fibers and the development of nanostructure in the bcc Ti matrix. The latter was associated with a decrease both in dislocation density from 4.0 × 10^15^ m^−2^ at N = 1 to 1.5 × 10^15^ m^−2^ at N = 5 and in the (sub)grain size from ~75 nm at N = 1 to ~50 nm at N = 5. A uniform chemical composition of the bcc Ti matrix was observed after 5 revolutions. 

(3) The microhardness of the Ti-15Mo/TiB MMC increased during HPT, reaching the maximum (730 HV) at the specimen edge after 5 turns. The precipitation hardening made the main contribution to the overall hardness. Meanwhile, substructure and gain-size (Hall–Petch) hardenings together had a ~2 times smaller contribution.

## Figures and Tables

**Table 1:** Parameters of Ti-15Mo/TiB metal-matrix composite (MMC) microstructure in the initial condition and after 1, 3 or 5 revolution of HPT calculated using XRD data.

Condition	Microstrain, $\langle \varepsilon_{50}^{2}\rangle$	Crystallite Size, D (nm)	Dislocation Density, ρ (10^15^ m^−2^)
Initial	0.0013	>200	0.28
N = 1	0.0049	49	4.0
N = 3	0.0037	33	2.3
N = 5	0.0030	21	1.5
